# Selection of effective LRAs using a newly designed *in vitro* HIV latency reactivation protocol: toward future application in HIV samples

**DOI:** 10.1128/spectrum.00407-26

**Published:** 2026-08-04

**Authors:** Alessia Neri, Arianna Rotili, Elena Morrocchi, Giuseppe Rubens Pascucci, Chiara Pighi, Giulio Olivieri, Federica Betto, Elena Emili, Ridika Karmakar, Daniela Iaconis, Filippo Lunghini, Gabriele Coppola, Donato Amodio, Nicola Cotugno, Paolo Palma

**Affiliations:** 1 PhD Program in Immunology, Molecular Medicine and Applied Biotechnology, University of Rome Tor Vergata 9318, Rome, Italy; 2 Clinical Immunology and Vaccinology Unit, IRCCS, Bambino Gesù Children's Hospital 9342 , Rome, Italy; 3 Probiomics S.r.l., Rome, Italy; 4 Department of Systems Medicine, University of Rome Tor Vergata 9318, Rome, Italy; 5 EXSCALATE, Dompé Farmaceutici SpA, Naples, Italy; 6 Chair of Pediatrics, Department of Systems Medicine, University of Rome Tor Vergata 9318, Rome, Italy; Penn State College of Medicine, Hershey, Pennsylvania, USA

**Keywords:** HIV cure, latency, latency reversal, *in vitro* models, computer-aided drug discovery

## Abstract

Human immunodeficiency virus type 1 (HIV-1) persists in latent reservoirs, mainly within CD4^+^ T cells, which are refractory to antiretroviral therapy, and can lead to rapid viral rebound upon treatment interruption. The “shock and kill” strategy aims to eliminate latent reservoirs by inducing viral transcription through latency-reversing agents (LRAs), thereby exposing infected cells to immune-mediated clearance. We developed and validated a simple, low-cost, and highly reproducible *in vitro* screening protocol to evaluate the efficacy and safety of LRAs, using a two-color flow cytometry assay on ACH2 cells, a well-characterized model of HIV-1 latency. Using this method, we identified PEP005 and CUDC-907 as the most potent LRAs across multiple experimental settings. Their reactivation capacity was further confirmed through transcriptomic analysis, which revealed a significant upregulation of viral RNA copies following stimulation. In addition, we collaborated with Dompé for using the Exscalate platform, an innovative computer-aided drug discovery approach, together with the experimental validation, which led to the identification of Tandutinib, a tyrosine kinase and mTOR inhibitor, as a novel LRA candidate with appreciable latency-reversing activity in the ACH2 model. While the ACH2 cell line does not fully recapitulate the complexity of HIV latency *in vivo*, it offers a robust and scalable system for early-stage screening and prioritization of candidate LRAs. Importantly, the future application of these LRAs in *ex vivo* samples derived from people living with HIV, with a particular focus on pediatric samples, will be crucial to deepen our understanding of latency reactivation in clinically relevant settings and age-specific immune environments.

**IMPORTANCE:**

This study addresses a major obstacle to curing human immunodeficiency virus type 1 (HIV-1) infection: the persistence of latent viral reservoirs that are not eliminated by current therapies. We developed a simple and reproducible assay to identify compounds capable of reactivating latent virus, a key step in cure strategies. Using this approach, we identified effective latency-reversing compounds and, through collaboration with Dompé using the EXSCALATE platform, we also identified Tandutinib as a promising new candidate for further investigation.

## INTRODUCTION

Human immunodeficiency virus type 1 (HIV-1) establishes latency by integrating its genome into the host DNA, mainly in CD4^+^ T cells, forming transcriptionally silent proviruses that persist despite antiretroviral therapy (ART). These latent reservoirs represent a major barrier to achieve a cure. Reactivation of these reservoirs can result in viral rebound following treatment interruption or virologic failure (1–5). Most strategies aimed at eliminating latently infected cells rely on the “shock and kill" approach (6). In this strategy, latency-reversing agents (LRAs) are used to induce proviral transcription (“shock”), which makes infected cells susceptible to immune-mediated clearance or cytopathic effects (“kill”) (4, 6–8). LRAs exert their effects by modulating various cellular pathways involved in the establishment and maintenance of HIV-1 latency (9, 10). Epigenetic modifications play a central role in regulating gene expression and represent key targets for reversing HIV-1 latency (11). In latently infected cells, the HIV-1 promoter harbors two CpG islands that are often hypermethylated, contributing to transcriptional silencing (12). Additionally, histone hypoacetylation promotes chromatin condensation, further repressing viral gene expression (11–14). Together, DNA methylation and histone deacetylation create a repressive chromatin environment that locks HIV-1 in a latent state, representing a significant challenge to eradication efforts (12). Several classes of LRAs act as epigenetic modulators and include inhibitors of DNA methyltransferase, histone deacetylase, and bromodomain and extra-terminal proteins (15–18). Other LRAs include protein kinase C (PKC) agonists that culminate in the NF-kB translocation to the nucleus (19), where it binds to the HIV long terminal repeat and recruits histone acetyltransferases that promote viral transcription by enhancing histone acetylation (19, 20). Aminobisphosphonates (N-BPs) are another class of LRAs that inhibit the enzyme farnesyl pyrophosphate synthase in the mevalonate pathway (21). It has been hypothesized that N-BPs exert a dual effect by inducing both HIV-1 reactivation and the activation of T cell cytotoxicity and adjuvant effects, which could lead to the elimination of HIV-1 reservoirs (21). Testing a large number of candidates LRAs directly in people living with HIV (PLWH) can be challenging, particularly in the pediatric setting, where investigation of HIV-1 latency is inherently limited by ethical and practical constraints, including the availability of biological samples. Moreover, in children who initiate ART very early after birth, the size of the replication-competent HIV reservoir can be extremely small and sometimes undetectable in *ex vivo* assays. Alongside the literature-based selection of molecules described as LRAs, we sought to expand the range of candidate compounds through a complementary *in silico* strategy. Repurposing clinically approved or advanced-stage compounds represents an attractive approach, as it enables the exploration of molecules previously approved in clinical trials with known pharmacokinetic and safety profiles that have not been previously classified as LRAs. To this end, in collaboration with Dompè, we employed the use of the Exscalate platform (22), a novel computer-aided drug discovery (CADD)-based approach to computationally prioritize candidate compounds for experimental evaluation. To efficiently validate these candidates, we established a robust and standardized experimental readout based on intracellular p24 detection by flow cytometry, which has previously been used to quantify HIV reactivation in cell line models and primary CD4^+^ T cells (23–25), as a functional endpoint rather than a standardized, dual-readout screening platform. In our study, we focused on the establishment of a simplified and reproducible two-color flow cytometry-based screening protocol in the well-characterized HIV-1 latently infected ACH2 T cell line. Indeed, our protocol simultaneously measures p24 and cell viability at single-cell resolution, providing a biologically meaningful quantitative and comparative assessment of reactivation while accounting for cytotoxicity. Importantly, it allows a transversal screening strategy that integrates literature-derived and computationally prioritized candidates within a unified experimental framework, thus providing an innovative, easy, reproducible, low-cost, and time-efficient approach. Finally, this protocol serves as a pre-screening platform to select a restricted panel of candidate LRAs for subsequent testing in PLWH-derived CD4^+^ T cells using distinct experimental workflows, including viral and host transcriptomic profiling. This stepwise strategy maximizes the efficient use of limited material, isolated from perinatally acquired HIV (PHIV) individuals, while supporting the integration of CADD with experimental validation in clinically relevant contexts.

## MATERIALS AND METHODS

### Selection of LRAs: literature-based and *in silico* identification of novel LRA candidates through polypharmacology prediction

A panel of LRAs was selected based on previous studies (16, 21, 26–30) reporting HIV-1 latency reversal in human *in vitro* models, including both latently infected cell lines and primary CD4^+^ T cells (all reagents and consumables information are reported in Table S1). Selection criteria included documented efficacy in inducing HIV-1 reactivation, evidence of activity in *ex vivo* or clinical studies where available, representation of compounds with differing degrees of novelty, and the availability of well-defined dosing information. For instance, the aim of representing both established and emerging molecules, rather than focusing exclusively on the most extensively studied LRAs, drove the selection of Apabetalone and CPI-203 as representative compounds of the BET inhibitor class. Practical considerations, including commercial availability and suitability for reproducible experimental testing, were also taken into account. To ensure biological relevance and experimental comparability, compound concentrations were chosen based on published cytotoxicity and efficacy data, prioritizing doses reported to be effective while exhibiting minimal cytotoxicity and closely matching those previously applied in comparable experimental systems. To account for the molecular heterogeneity of HIV-1 latency, LRAs were chosen from diverse pharmacological classes, targeting distinct cellular pathways involved in the maintenance of viral latency (Table 1). To further expand the pool of molecules with potential latency-reversing activity, we employed the Exscalate platform (22), which leverages a comprehensive compound library curated from various studies and integrates CADD approaches that have become increasingly valuable in drug discovery pipelines due to advances in supercomputing capabilities. For this study, we used two modules of the Exscalate platform: ProfhEX and EXDII. ProfhEX (31) is a suite of machine learning models trained on over 5 million bioactivity data points to provide high-confidence potency predictions across 693 human protein targets. This large-scale predictive framework allows for the comprehensive annotation of compound libraries with *in silico*-driven polypharmacology, facilitating the identification of secondary interactions beyond primary mechanisms. EXDII is a proprietary Exscalate tool devoted to automatic integration of comprehensive up-to-date drug information collected from public and private databases (Cortellis Drug Discovery Intelligence by Clarivate (32); DrugCentral (33, 34), and ChemBL (35, 36). Using the ProfhEX module, we conducted a comprehensive polypharmacology profiling of PEP005 and CUDC-907, selected as the best molecules in our first experiments, systematically annotating all potential protein targets to identify complementary mechanisms that may contribute to latency reversal (Fig. S1A). Following, we took advantage of EXDII for a target expansion approach. This tool annotates all the drugs that can modulate the targets identified in the previous step (Fig. S1B). Polypharmacology analysis enabled the identification of novel protein families, which guided the selection of new drugs for potential repurposing among the ones with top ranking (Table 2). These selected compounds are either in preclinical development or already approved for clinical use for unrelated indications, and we tested them alongside PEP005 and CUDC-907.

**TABLE 1:** Selected LRAs molecules with their mechanism of action and effective doses

Molecule	Class	Concentration	Reference
Apabetalone	BET bromodomain inhibitor	30 uM	(26)
Alendronate	Aminobisphosphonates, N-BPs	2.5 uM	(21)
5-Aza-2′-deoxycytidine (5-AzadC)	DNA methylation inhibitor	1 uM	(16)
CPI-203	BET bromodomain inhibitor	1 uM	(27)
(−)-Epigallocatechingallate (EGCG)	DNA methylation inhibitor	70 ug/mL	(28)
Ingenol-3-angelate (PEP005)	PKC agonist	0.012 uM	(29)
Fimepinostat (CUDC-907)	Histone deacetylases and PI3K inhibitor	25 nM	(30)

**TABLE 2:** Molecules predicted by the *in silico* polypharmacology-driven identification to modulate HIV latency reversal pathways, with their proposed mechanisms of action, current clinical indications, and tested concentration

Molecule	Class	Tested concentration	Approved for	Reference
Bexarotene	Retinoid acid receptor (RXR) agonists	10 uM, 25 uM	Cutaneous T cell lymphoma (CTCL)	(37)
Tamibarotene	Retinoid acid receptor (RXR) agonists	10 uM, 25 uM	Acute promyelocytic leukemia	(38)
Tandutinib	(PI3K)/Akt and mTOR inhibitor	10 uM, 25 uM	Acute myeloid leukemia	(39)
Triclabenzadole	DNA methyltransferase 1 (DNMT1) inhibitors	10 uM, 25 uM	Chronic fascioliasis	(40)
Vorinostat	HDAC inhibitor	350 nM, 400 nM	Cutaneous T cell lymphoma (CTCL)	(41)

#### Culture and maintenance of HIV-1 latently infected ACH2 and 8E5LAV cell lines

We initially tested LRAs on two different HIV-bearing cell lines: ACH2 and 8E5LAV (42). The ACH2 cell line is derived from human acute lymphoblastic leukemia T cells and harbors a single integrated copy of HIV-1 (strain LAI). These cells are replication-competent and carry a mutation in the Tat responsive element stemloop region of the HIV-1 LTR, which affects Tat-mediated transcriptional regulation (43). ACH2 cells display reduced CD4 surface expression and resistance to superinfection by the same HIV-1 strain, features commonly associated with chronically infected T-cell lines (44, 45).

The 8E5LAV cell line (46, 47), also derived from human lymphoblastoid leukemia cells, contains a single integrated, replication-defective provirus, due to a mutation in the *pol* gene. These cells continuously produce non-infectious viral particles and are commonly used as a quantitative standard for measuring the frequency of latently infected HIV-1 reservoir cells. Both cell lines were thawed in complete RPMI 1640 medium (cRPMI), supplemented with 10% heat-inactivated FBS, 1% L-glutamine, and 1% Pen/Strep, seeded, and expanded into tissue culture flasks. Cultures were maintained at 37°C, 5% CO_2_, for a maximum of two consecutive months, refreshing medium every 2–3 days. Cell morphology, viability, and potential contamination were regularly monitored using an optical microscope and routinely tested for mycoplasma contamination. Both cell lines were obtained from the Center for AIDS Reagents.

### Blood processing and peripheral blood mononuclear cell isolation

Peripheral blood samples were collected from healthy donors via venipuncture into EDTA-coated tubes (BD Vacutainer) and processed within 2–4 h. Samples were first centrifuged to separate the plasma, which was then stored at −80°C. Peripheral blood mononuclear cells (PBMCs) were isolated by Ficoll-Paque PLUS density gradient centrifugation, washed twice with PBS, and counted to assess the viability using the Countess II automated cell counter. PBMCs were then cryopreserved in 1 mL of cold Freezing medium (90% FBS, 10% dimethyl sulfoxide, DMSO), initially stored at −80°C in a controlled-rate freezing container, and subsequently transferred to liquid nitrogen until use.

### Isolation of CD4^+^ T cells from PBMCs

PBMCs were thawed at 37°C in a water bath and immediately transferred dropwise into pre-warmed cRPMI. CD4^+^ T cells were enriched from PBMCs by negative selection using the EasySep Human CD4^+^ T Cell Enrichment Kit according to the manufacturer’s instructions. Briefly, PBMCs were adjusted to a concentration of 50 × 10^6^ cells/mL, and the EasySep Enrichment Cocktail was added to the cell suspension at a concentration of 50 µL/mL, gently mixed, and incubated for 10 min at RT. Next, EasySep Magnetic Particles were vortexed thoroughly and added to the cell suspension at 100 µL/mL, followed by a second 10-min incubation at RT. During this time, a recommended medium (PBS supplemented with 2% FBS and 1 mM EDTA) was prepared and added to the tubes, reaching 2.5 mL. The tubes were then placed in the EasySep magnet and left undisturbed for 5 min at RT to allow magnetic separation. The enriched CD4^+^ T cells that remained in suspension were carefully collected, without disturbing the magnetic particles, washed twice with PBS, and resuspended in cRPMI. A final cell count was performed before proceeding with downstream stimulation protocols.

### *In vitro* stimulation with LRAs

An experimental protocol was established to assess the latency-reversing activity of selected LRAs under various stimulating conditions, including both positive and negative controls. ACH2 and 8E5LAV cell lines, as well as isolated CD4^+^ T cells, were counted and seeded in round-bottom 96-well plates at a density of 0.5–1 × 10^6^ cells/well (with adjustments based on cell growth rate and confluency), in a final volume of 250 µL of cRPMI supplemented with 400 nM Azidothymidine (AZT; 3′-azido-3′-deoxythymidine) to prevent viral spread (48). Cells were stimulated with individual LRAs at different concentrations selected from previously published studies or *in silico* predictions (Tables 1 and 2, respectively). Unstimulated condition (cRPMI + AZT alone) served as a negative control, while a combination of phorbol 12-myristate 13-acetate (PMA, final concentration 81 nM), ionomycin (final concentration 1.34 µM), and interleukin-2 (IL-2, final concentration 20 U/µL) was added to the culture medium, serving as a positive control. Each condition was tested in triplicate and incubated at 37°C in a humidified 5% CO₂ incubator for 20, 48, or 72 h. After stimulation, cells were harvested for viability assessment and intracellular p24 staining to evaluate HIV-1 reactivation. Initially, for the purpose of selecting the most appropriate positive control according to stimulation timing, we evaluated several reagents acting as inducers of HIV-1 reactivation. In specific, we tested three distinct conditions: (i) PMA (5 ug/mL) + PHA (5 ug/mL), selected on a previously published protocol (33); (ii) PMA (81 nM) + ionomycin (1.34 µM) + IL-2 (20 U/µL), previously validated by our group for 20 h stimulation (49); (iii) PMA alone (25 nM), commonly reported in literature for prolonged stimulation protocols (48–72 h) (30). Among these, since the PMA/ionomycin/IL-2 combination was the most effective positive control in our assay, at all timing tests, it was selected for our protocol (data not shown).

### p24 and viability detection by flow cytometry

Following stimulation, cells were stained and then washed once with FACS buffer (FB, PBS supplemented with 1% FBS). Viability staining was performed using BD Horizon Fixable Viability Stain 510 (FVS510) at a final concentration of 1 µL/mL in FB and incubated for 15 min at RT in the dark. After incubation, cells were washed once with FB to remove excess dye. Subsequently, cells were fixed by adding 100 µL of fixation buffer (PBS containing 1% paraformaldehyde, PFA) and incubated for 20 min at RT. After an additional wash with FB, permeabilization was performed by incubating the cells for 20 min at RT with 100 µL of BD Perm Solution II, diluted 1:10 in distilled water. Intracellular p24 staining was performed using anti-p24-PE antibody. For each tube, 3 µL of antibody was added to 47 µL of BD Perm Buffer, mixed thoroughly, and incubated for 30 min at 4°C, protected from light. Lastly, cells were washed and resuspended in 200 µL of FB. Data were acquired using a CytoFLEX flow cytometer (Beckman Coulter Life Sciences), and analysis was performed using appropriate gating strategies to assess both p24 expression and cell viability, with the FlowJo software (v10).

### RNA isolation

Stimulated ACH2 cells were collected and resuspended in 350 µL RNeasy Lysis Buffer supplemented with 10 µL/mL of β-mercaptoethanol. Lysates were stored overnight at −80°C for at least 1 day. Then, total RNA was extracted using the RNeasy Micro Kit or Mini Kit, following the manufacturer’s protocol. RNA was eluted in 14 or 30 μL of RNase-free water, depending on the used kit, and 1 μL of RNase inhibitor was added to prevent RNA degradation. RNA concentration (ng/μL) was measured using the Qubit RNA HS Assay Kit and Qubit Fluorometer. Final RNA samples were stored at −80°C until further use.

### HIV copies detected through RNA sequencing

RNA sequencing was carried out using the long-read platform provided by Oxford Nanopore Technologies. The PCR-cDNA Barcoding Kit (SQK-PCB109) was used to prepare the library, following the manufacturer’s instructions. Briefly, starting from 50 ng of full-length RNA, complementary DNA (cDNA) was synthesized, followed by strand-switching using kit-specific oligonucleotides. The cDNA was amplified by PCR (10 μL per sample) to generate double-stranded cDNA (dscDNA), using primers that included 5′ tags to allow direct, ligase-free attachment of Rapid Sequencing Adapters. Libraries were loaded onto R9.4.1 flow cells (FLO-MIN106) for sequencing or stored at −80°C until further use. Sequencing was performed using MinION devices with MinKNOW software (v21.02.01).

### Data analysis

Flow cytometry data were analyzed by FlowJo software (v10), calculating the mean fluorescence intensity (MFI) of the p24 marker and the frequency (%) of live cells to measure viability. GraphPad Prism (v10.4.1 627) was used for both statistical analysis and graphical representation. In Dotmatics and Histogram plots, data were plotted as mean ± standard deviation (SD). Sequencing FAST5 files was basecalled with the standalone Guppy basecaller (v.6.2.11). Adapters and barcodes were trimmed during demultiplexing, which was performed using Guppy barcoder (v6.2.11). The quality of the reads was checked with the fastqc tool (v.0.11.9), and the reads with more than 40% of the bases with a Phred score of less than 10 were filtered with fastp (v.0.12.4). Next, the high-quality reads were aligned against hg38 Gencode human transcriptome reference (v38) using minimap2 (v.2.21) with map-on preset parameters. Reads unmapped on the hg38 transcriptome were aligned against the HIV-1 (HXB2) genome sequence (GenBank: K03455.1) using minimap2 with the splice preset parameters. The quantification was calculated as (number of HIV reads/number of high-quality reads) × 10^6^.

## RESULTS

### Establishment of a standardized *in vitro* framework to evaluate HIV-1 latency reversal

**Fig 1:**
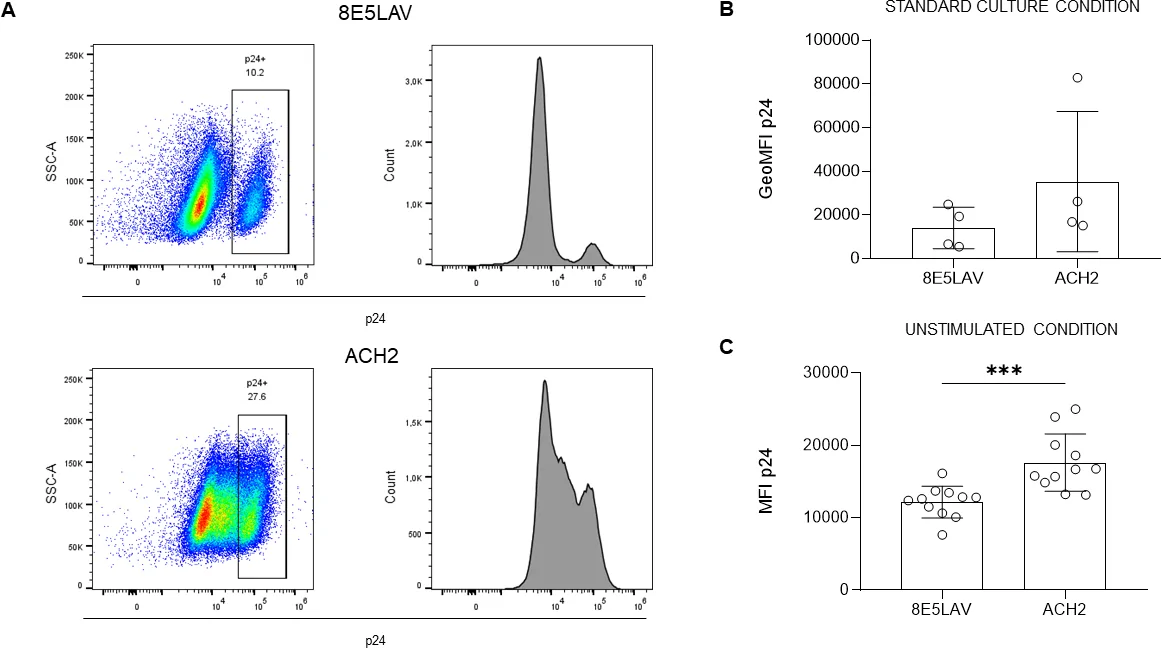
Comparison of p24 expression levels between the two cell lines. Comparison between the basal levels of p24 in the 8E5LAV and ACH2 cell lines. (**A**) p24 frequency (left) and cell count (right) of live 8E5LAV (above) and ACH2 (below) cell lines are reported. (**B**) Comparison between basal p24 GeoMFI of the two cell lines in standard culture conditions (*n* = 3). (**C**) Comparison between p24 expression in terms of GeoMFI in the unstimulated condition after 20, 48, and 72 h in the plate, *n* = 11. Mann-Whitney test was applied (* = *P* value < 0.05, ** = *P* value < 0.01, *** = *P* value < 0.001, absence of an * indicates no significant differences) for comparisons between cell lines.

**Fig 2:**
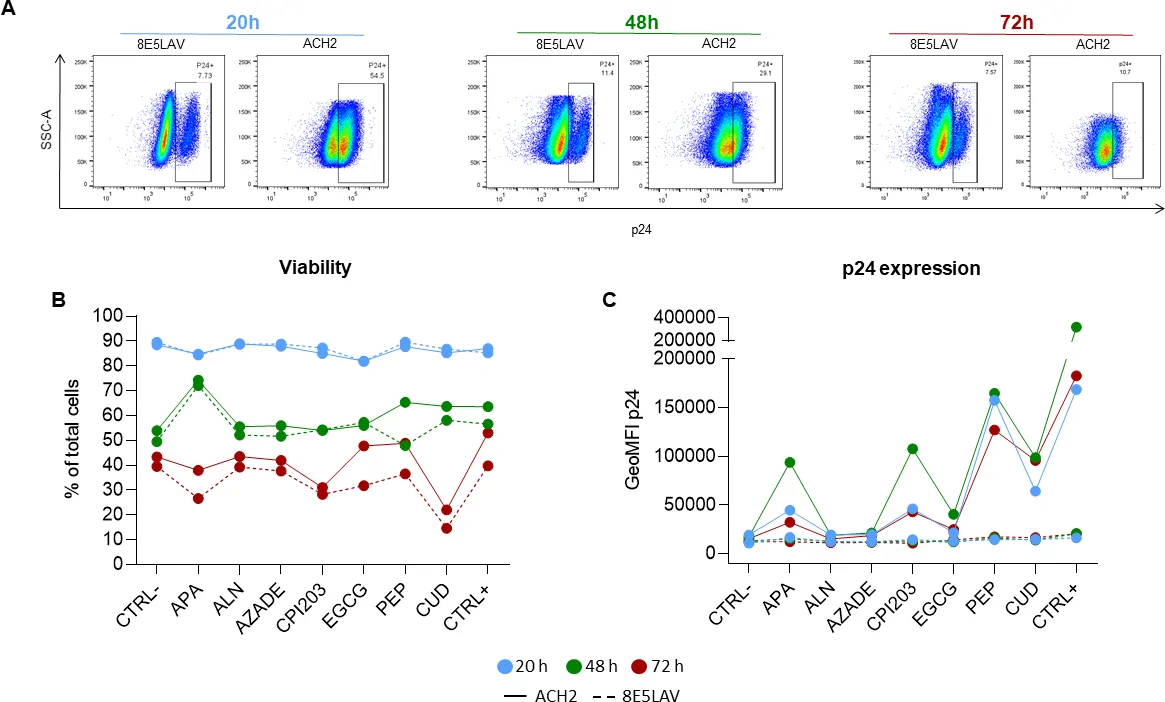
Comparisons between ACH2 and 8E5LAV and the three (20, 48, and 72 h) timings of stimulation. (**A**) Exemplary gating strategy on the Alendronate molecule shows the shift of the p24 gate between the ACH2 and 8E5LAV cell lines and at different times of stimulation. (**B**) Frequencies of live cells on the total number at all different conditions of stimulation and after the three timings of stimulation. *N* = 4. (**C**) GeoMFI of the p24 expression on live cells at all different conditions and after the three timings of stimulation is reported. The continuous line and dashed line represent the ACH2 and 8E5LAV cell lines, respectively. In light blue, we reported the results after 20 h of stimulation, in green, the 48 h, and in red, the 72 h of stimulation. *N* = 4. No statistical significance is reported in the figure.

The overall aim of this study was to develop a standardized and reproducible protocol to compare the activity of distinct LRAs using *in vitro* models of HIV-1 latency, with the specific goal of enabling an effective prescreening of compounds prior to testing in primary cells. The assay involved the stimulation of HIV-1-infected cell lines with selected LRAs, followed by the quantification of viral reactivation through intracellular p24 expression measured by flow cytometry. Cell viability was simultaneously assessed, using viability staining, to evaluate compound-associated cytotoxicity. Both reactivation capacity and viability were used as key indicators of LRA performance, balancing efficacy with safety. To strengthen the robustness of the approach, two well-established HIV-1-infected cell lines, ACH2 and 8E5LAV, were systematically evaluated and compared throughout the optimization process. To identify the most suitable cellular model for our needs, we compared the two cell lines for their baseline p24 expression and responsiveness to the selected LRA panel. ACH2 cells exhibited higher p24 expression than 8E5LAV cells in standard culture conditions (Fig. 1A and B), although the difference was not statistically significant. However, in the unstimulated conditions following *in vitro* incubation, the ACH2 cells showed significantly higher p24 levels (*P* = 0.0003), measured as geometric mean fluorescent intensity (GeoMFI), than the 8E5LAV cells (Fig. 1C). To identify the optimal stimulation timing, we assessed p24 expression and cell viability at 20-, 48-, and 72-h post-stimulation with seven distinct LRAs (Table 1). Figure 2 presents the results in terms of live cell frequency and p24 GeoMFI after *in vitro* stimulation under the indicated conditions, for both cell lines. Importantly, cell viability showed a highly consistent pattern across both cell lines, displaying comparable trends in response to different LRAs and across stimulation time points. These results indicate that both the nature of the compounds and the duration of stimulation exert a major and reproducible impact on cellular viability, independently of the specific latency model used. The timing of 72 h of stimulation demonstrated a drop of viability, with all conditions showing less than 55% viability (Fig. 2B), without any advantage in terms of p24 expression compared to the shorter time points (Fig. 2C). For this reason, the 72 h timing was excluded in the final protocol.

### Selection of the optimal cellular model and stimulation time for LRA prescreening protocol

**Fig 3:**
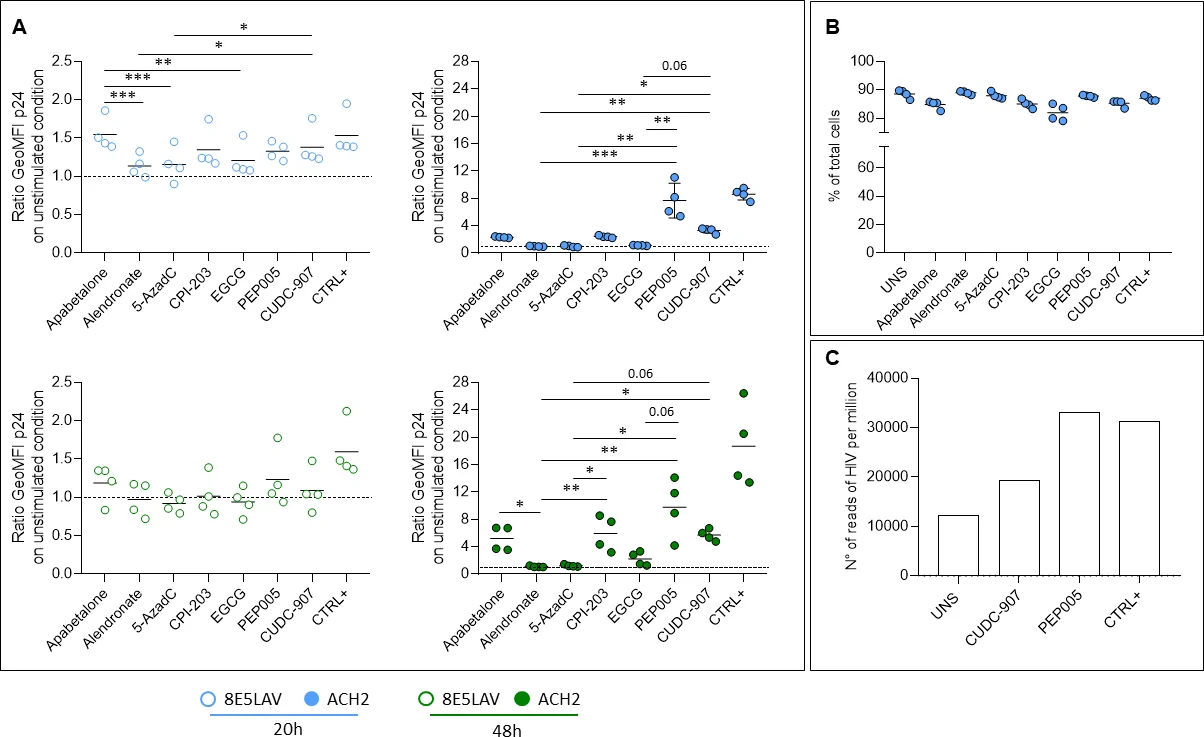
Selection of the cell lines and time of stimulation and of the best LRAs. Comparison between the different molecules on the ACH2 and 8E5 cell line after 20 h and 48 h of stimulation. (**A**) Measurement of the potential of reactivation as a ratio (stimulated condition/unstimulated condition) of p24 GeoMFI on the live cells for both 8E5LAV and ACH2, for 20 and 48 h of stimulation. Only significant differences between the molecules are reported, excluding those with the positive control. *N* = 4, Friedman test and uncorrected Dunn’s test (* = *P* value < 0.05, ** = *P* value < 0.01, *** = *P* value < 0.001, absence of an * indicates no significant differences). (**B**) Frequencies of live cells on the total number of cells at all different conditions of stimulation after 20 h of stimulation on the ACH2 cell lines. *N* = 4, Friedman test and uncorrected Dunn’s test (* = *P* value < 0.05, ** = *P* value < 0.01, *** = *P* value < 0.001, absence of an * indicates no significant differences). (**C**) Number of reads per million that aligned to the reference HIV viral genome HBX2 revealed RNA sequencing using Nanopore technology after stimulation with PEP005, CUDC-907, and positive control compared to the unstimulated condition.

To better contextualize reactivation levels, p24 expression was normalized to unstimulated conditions and expressed as a ratio for both cell lines. In 8E5LAV cells, although the 20-h time point consistently demonstrated some significant differences between the molecules compared to 48 h, the magnitude of reactivation remained limited, with ratios rarely exceeding 1.5 across all conditions, including the positive control for both the timing of stimulation (Fig. 3A and B). Despite some significant differences being present at 20-h (Fig. 3A, upper left, Apabetalone vs Alendronate *P* = 0.0005, Apabetalone vs 5-AzadC *P* = 0.0009, Apabetalone vs EGCG *P* = 0.0024, Alendronate vs CUDC-907 *P* = 0.03, 5-AzadC vs CUDC-907 *P* = 0.043), such a narrow dynamic range, particularly when using a flow cytometry-based readout, does not allow sufficient discrimination of compound-specific reactivation capacity. For this reason, despite their stability, 8E5LAV cells were considered suboptimal for protocol setup and downstream screening purposes. In contrast, ACH2 cells displayed a broader and more informative reactivation range, exhibiting noticeably higher p24 levels variation than 8E5LAV. Given the clearer discrimination of compound-specific effects on reactivation in ACH2 cells, they were selected as models for further analyses. A clear trade-off between reactivation efficacy and cell viability emerged when comparing the two stimulation time points. At 20 h, only two compounds (PEP005 and CUDC-907) showed significant latency-reversing activity (Fig. 3A, upper right, Alendronate vs PEP005 *P* = 0.0009, Alendronate vs CUDC-907 *P* = 0.009, 5-AzadC vs PEP005 *P* = 0.001, 5-AzadC vs CUDC-907 *P* = 0.01, EGCG vs PEP005 *P* = 0.009), while preserving high cell viability (approximately 80%–90%, Fig. 2B). Extending the stimulation to 48 h increased the number of effective LRAs to four (Fig. 3A, down right, Apabetalone vs Alendronate *P* = 0.02, Alendronate vs CPI-203 *P* = 0.009, Alendronate vs PEP005 *P* = 0.002, Alendronate vs CUDC-907 *P* = 0.01, 5-AzadC vs CPI-203 *P* = 0.04, 5-AzadC vs PEP005 *P* = 0.01, 5-AzadC vs CUDC-907 *P* = 0.06, EGCG vs PEP005 *P* = 0.06), with the BET inhibitors Apabetalone and CPI-203 displaying enhanced reactivation, in line with published evidence supporting their time-dependent activity (26, 27). Importantly, although at 48 h of stimulation additional BET inhibitors reached statistical significance, the overall level of reactivation achieved by these compounds remained lower than that observed for PEP005 and CUDC-907. This was reflected by a lower number of significant pairwise comparisons, indicating that PEP005 and CUDC-907 consistently outperformed the other molecules at both 20 and 48 h. Accordingly, despite the emergence of additional significant compounds at the later time point, PEP005 and CUDC-907 remain the best-performing molecules also at 48 h. Moreover, PEP005 and CUDC-907, already selected as the most effective molecules at 20 h, were confirmed to induce the highest levels of reactivation at 48 h, albeit at the expense of a substantial reduction in cell viability, which decreased to approximately 70%–65%. Taken together, these data indicate that although prolonged stimulation (48 h) identifies additional candidate molecules, the compounds showing the most robust and consistent reactivation across time points are PEP005 and CUDC-907. In line with the primary objective of establishing a prescreening strategy to prioritize compounds for subsequent testing in primary CD4^+^ T cell samples, the 20-h stimulation consistently identified the most effective LRAs while preserving a substantially higher proportion of viable cells. While we acknowledge the trade-off between reactivation magnitude and cell viability, this protocol does not preclude further testing using longer stimulation times to define optimal reactivation conditions. Based on this balance between reactivation capacity and cell viability, the 20-h time point was selected as the optimal condition for the standardized *in vitro* prescreening assay.

### Identification of the top-performing LRAs

Based on the considerations mentioned before, the established protocol was used to evaluate which of the selected molecules demonstrated the strongest latency-reversing potential. After 20 h of stimulation, all treatment conditions maintained good cell viability (above 80%), indicating that all compounds met the criteria for acceptable toxicity (Fig. 3B). As mentioned before, PEP005 and CUDC-907 demonstrated significantly higher reactivation potential compared to EGCG, 5-AzadC, and Alendronate (Fig. 3A, upper right), being the most effective LRAs among the seven compounds tested. To gain further insight into their latency-reversing activity, we assessed viral reactivation in the ACH2 cell line using Nanopore sequencing after stimulation with PEP005, CUDC-907, and the positive control. Alignment to the reference HIV genome (HBX2) revealed that PEP005 treatment resulted in the highest number of viral reads (~33,000 per million), followed by the positive control (~31,000 per million), CUDC-907 (~19,000 per million), and the negative control (~12,000 per million). These results confirm an increase in HIV viral copies following stimulation with the two selected compounds (Fig. 3C).

### Evaluation of the potential enhancement of latency reactivation by combining PEP005 and CUDC-907 with previously tested compounds

**Fig 4:**
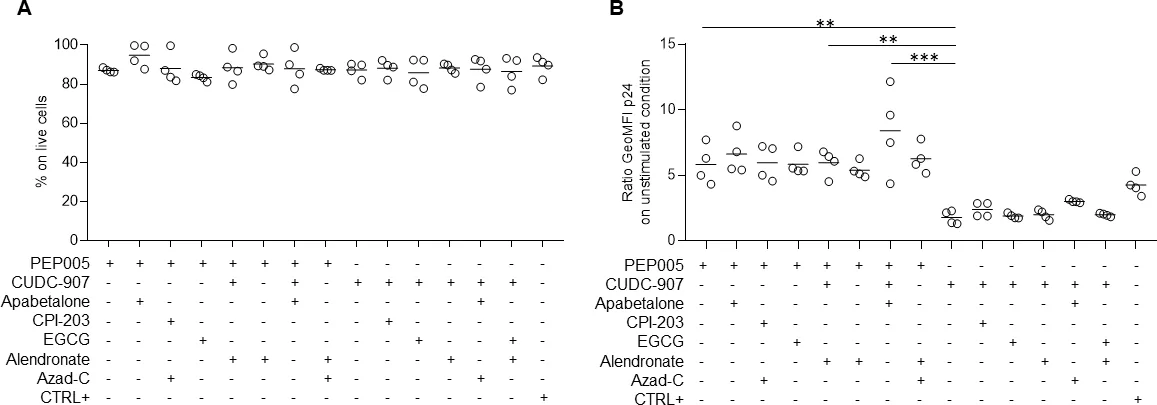
Comparison of efficacy between the molecules PEP005 and CUDC-907 and the combinations of different molecules. Comparison between PEP005 and CUDC-907 and the 12 combinations of molecules selected. (**A**) Frequency of live cells among the total number of cells after the stimulation under all the different conditions. (**B**) Measurement of the potential of reactivation as a ratio (stimulated condition/unstimulated condition) on GeoMFI on the p24 protein on the live cells at all the different conditions. In figures are reported the significant differences only between PEP005 and CUDC-907 used alone and their combinations, excluding the one with the positive control. *N* = 4, Friedman test and uncorrected Dunn’s test (* = *P* value < 0.05, ** = *P* value < 0.01, *** = *P* value < 0.001, absence of an * indicates no significant differences).

We investigated whether combining different LRAs could enhance their reactivation efficacy, aiming to assess potential additive effects. Given the large number of possible combinations, we implemented a systematic selection strategy to identify a representative set for further testing. Specifically, PEP005 and CUDC-907 were tested both individually to evaluate the effects of combination versus single-agent treatment, and in combination with other LRAs belonging to distinct pharmacological classes to maximize the likelihood of uncovering synergistic mechanisms. For each class, a single representative compound was selected to avoid redundancy. This approach resulted in a panel of 12 distinct combinations, allowing us to maintain experimental feasibility while exploring relevant interactions. All combinations showed good cell viability, with an average live cell frequency above 80% (Fig. 4A). Combinations, including PEP005, showed p24 expression levels comparable to the molecule alone, with no statistically significant differences, suggesting a lack of latency reactivation enhancement. In contrast, two combinations containing CUDC-907 (PEP005 + Apatabelatone + CUDC-907 and PEP005 + Alendronate + CUDC-907) exhibited significantly higher p24 expression compared to CUDC-907 alone (*P* = 0.0004 and *P* = 0.0053). Nevertheless, these effects appear primarily driven by the presence of PEP005, as the p24 levels were similar to those observed with PEP005 alone (Fig. 4B). This evaluation did not reveal any increase in reactivation potential for the tested combinations, supporting the decision to proceed with further analyses using PEP005 and CUDC-907 alone. To strengthen this result, we apply the highest single agent (HSA) model (50, 51), which is particularly suitable for experimental designs in which one compound dominates the biological response while other compounds show limited or no activity on their own. HSA scores were calculated as the difference between combination and single-agent effects, with positive values indicating improved activity of the combination. Statistical significance was assessed using a one-sided Wilcoxon signed-rank test. HSA values were generally small and centered around zero across all tested combinations. None of the tested conditions reached statistical significance under the HSA framework, confirming the previous analysis (data not shown).

### *In silico* polypharmacology-driven identification of candidate latency-reversing agents

**Fig 5:**
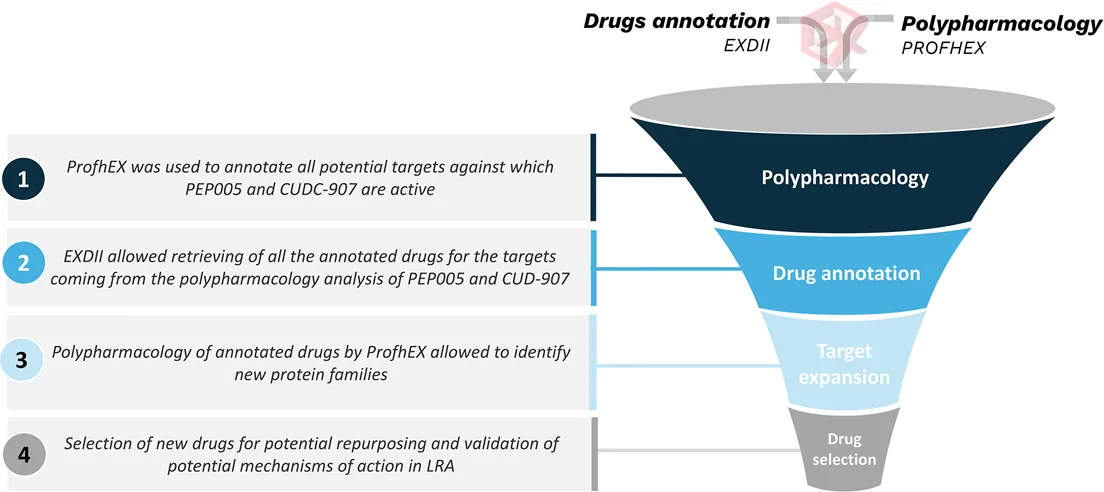
*In silico* workflow to enable new drugs for LRA. Overview of the computational pipeline implemented through the Exscalate platform. The workflow integrates ProfhEX and EXDII tools for polypharmacology and drug annotation analyses to identify new protein families and select candidate drugs for potential repurposing as LRAs.

To further expand the pool of molecules with potential latency-reversing activity, we employed the Exscalate platform (22), an advanced high-performance computing system for CADD. Exscalate platform leverages a comprehensive compound library curated from various studies (52, 53) and integrates CADD approaches that have become increasingly valuable in drug discovery pipelines due to advances in supercomputing capabilities. For this study, we used two modules of the Exscalate platform: ProfhEX and EXDII. The *in silico* workflow for the selection of the new compounds is reported in Fig. 5. By ProfhEX, a module of the platform, we computed predicted polypharmacology profiles of both PEP005 and CUDC907, the molecules selected previously as more efficient, systematically annotating all potential protein targets against which these compounds could exhibit an effect (at least in the micromolar range). This process resulted in a comprehensive compound-target bioactivity matrix, and we hypothesized that among all the annotated targets, some of them could be potentially responsible for latency reversal (Fig. 5, box 1 and Fig. S1A). We then queried the EXDII tool to retrieve a library of approved and investigational drugs known to modulate these proteins (Fig. 5, box 2 and Fig. S1B). To further uncover protein families functionally related to our initial set, we re-profiled these EXDII-retrieved drugs through ProfhEX (Fig. 5, box 3 and Table 2). This secondary expansion highlighted key families, such as the retinoid acid receptor (RXR) family, (PI3K)/Akt and mTOR pathway, and DNA methyltransferase (DNMT) and histone deacetylases (HDAC) families (Table 2). Finally, we ranked the drugs found by EXDII, which showed activity on those proteins/families (see Materials and Methods). This *in silico* analysis indicates which drugs should be tested against the selected mechanisms of action, proposing potential drugs for repurposing. In particular, Bexarotene and Tamibarotene as RXR modulators, Tandutinib as a PI3K/mTOR signaling modulator, Triclabendazole as a DNMT1 tool compound, and Vorinostat as an HDAC inhibitor (37–41) (Table 2). Notably, Vorinostat is a well-established latency-reversing agent, previously tested in experimental systems and clinical trials (54, 55).

### PEP005 AND CUDC-907 confirmed as the most effective LRAs, with Tandutinib emerging as a potential candidate among *in silico*-selected compounds

**Fig 6:**
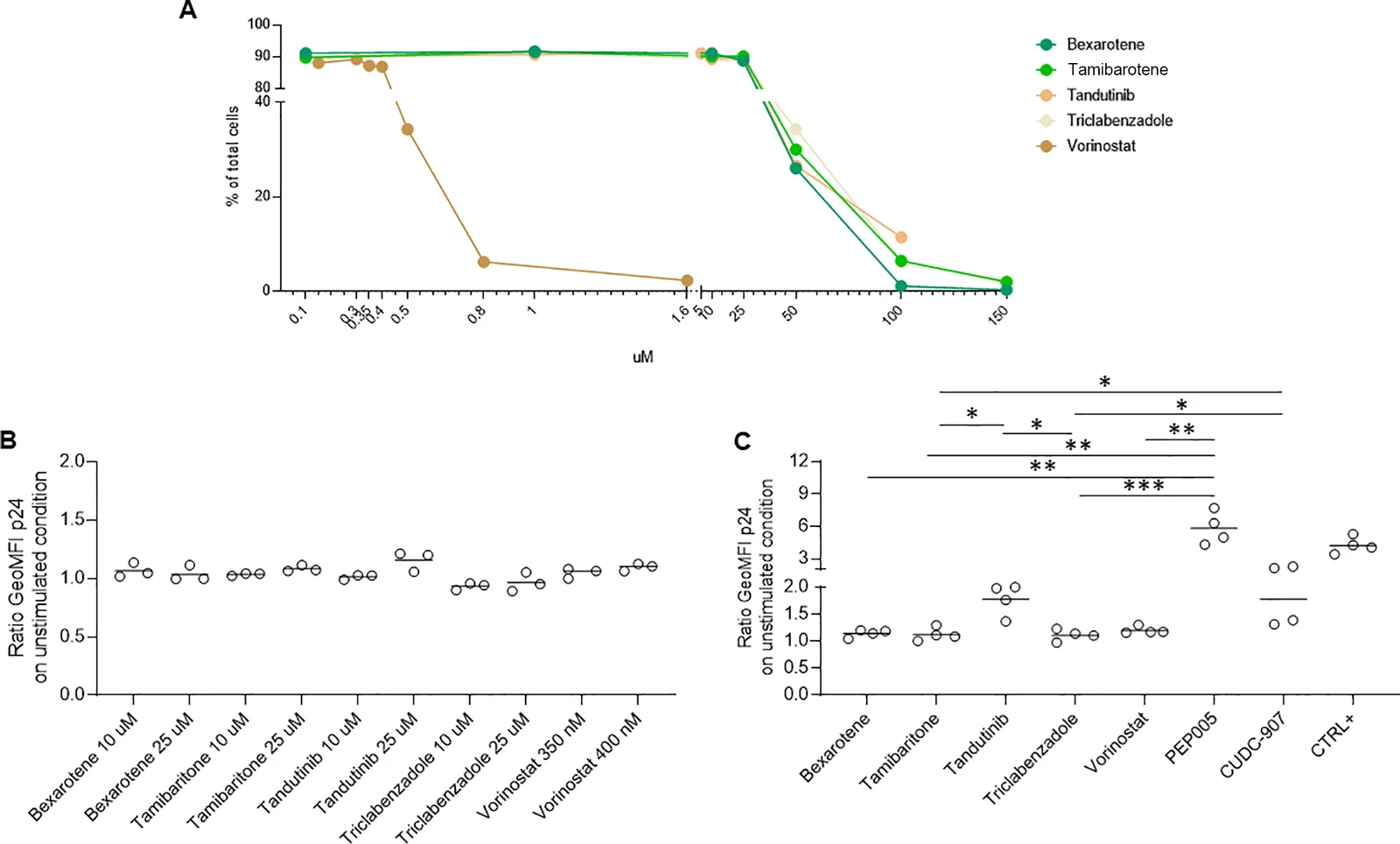
Selection of the best concentration for the *in silico* selected molecules and comparison of efficacy between the molecules PEP005 and CUDC-907, and novel molecules selected *in silico.* Selection of concentration for testing novel molecules identified with the *in silico* screening. (**A**) Viability curves were generated for the five novel compounds by measuring cell viability across a range of concentrations, in order to identify the threshold at which viability significantly declines. (**B**) Reactivation potential assessed as ratio (stimulated condition/unstimulated condition) of p24 GeoMFI in live cells following stimulation with two selected concentrations of each compound. Comparison between PEP005 and CUDC-907 and the novel molecules selected by the Exscalate platform. (**C**) Comparison between PEP005 and CUDC-907 and the novel molecules selected by the Exscalate platform. Measurement of the HIV reactivation potential is measured as p24 GeoMFI ratio (stimulated condition/unstimulated condition) on live cells at all the different conditions, excluding the ones with positive control. *N* = 4, Friedman test and uncorrected Dunn’s test (* = *P* value < 0.05, ** = *P* value < 0.01, *** = *P* value < 0.001, absence of an * indicates no significant differences).

These compounds were subsequently tested alongside PEP005 and CUDC-907. As they had previously studied different disease models and cell types, no standardized concentrations were available for our experimental setup. To identify appropriate conditions for HIV-1 latency reversal assays, we first performed dose-response titrations to assess cytotoxicity. Concentration ranges were selected based on Exscalate data, enabling us to define viability thresholds and generate dose-viability curves for each compound. We tested seven concentrations per compound using the ACH2 cell line and assessed cell viability after 20 h of stimulation. Bexarotene, Tamibarotene, Tandutinib, and Triclabendazole all exhibited a drop in viability below 40% at concentrations above 25 uM, while Vorinostat showed toxicity at concentrations above 400 nM (Fig. 6A). We then selected the two highest concentrations for each molecule that maintained viability above 80% and assessed p24 expression. As no significant differences were observed between the two concentrations for any compound in terms of both p24 expression ratio (Fig. 6B), we proceeded with the highest viable concentration for comparative analysis. When comparing the *in silico*-selected compounds, PEP005 and CUDC-907 demonstrated superior latency-reversing activity. Specifically, PEP005 showed significantly higher p24 expression than Triclabendazole, Bexarotene, Tamibarotene, and Vorinostat (ratio comparisons: *P* = 0.0005, *P* = 0.0039, *P* = 0.0005, *P* = 0.0039, respectively). CUDC-907 also showed significantly greater activity than Tamibarotene and Triclabendazole (ratio *P* = 0.043 for both comparisons). However, neither PEP005 nor CUDC-907 displayed enhanced latency reversal capacity compared to Tandutinib. Notably, Tandutinib exhibited significantly higher activity than Triclabendazole and Tamibarotene in terms of ratio (*P* = 0.043 for both comparisons). Although none of the *in silico*-predicted compounds surpassed CUDC-907 or PEP005 in latency reversal potency, Tandutinib emerged as a promising candidate with potential of latency-reversing activity, warranting further investigation validating the proposed *in silico* approach for repurposing (Fig. 6C).

### PEP005 AND CUDC-907 are not cytotoxic for primary CD4^+^ T cells

**Fig 7:**
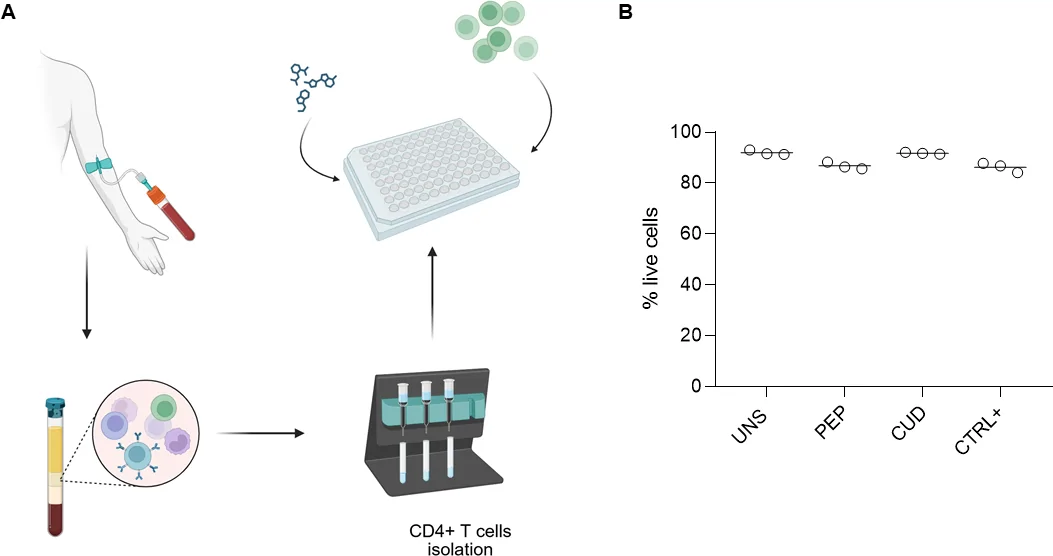
Toxicity of PEP005 and CUDC-907 on CD4^+^CD4^+^ T cells. (**A**) Illustrative schematic of the experimental workflow for LRA stimulation on primary patient-derived cells, from peripheral blood collection to PBMC isolation and subsequent CD4+ T cell isolation. (**B**) Frequency of live cells on the total cells after stimulation with PEP005, CUDC-907, and positive control, measured by flow cytometry on CD4^+^ T cells isolated from PBMCs obtained from healthy donors. *N* = 3.

To assess the potential cytotoxicity of the selected LRAs on primary cells, we tested PEP005 and CUDC-907 on CD4^+^ T cells isolated from PBMCs of healthy donors (Fig. 7A). Given that cell lines and primary cells may respond differently to stimulation, we applied the same protocol established for the cell line assays and evaluated cell viability by flow cytometry. Following 20-h stimulation, both compounds preserved high levels of viability in CD4^+^ T cells, with live cell frequencies consistently exceeding 80% (Fig. 7B). These results support the suitability of PEP005 and CUDC-907 for *in vitro* studies involving primary human T cells.

## DISCUSSION

The mechanisms that govern HIV latency establishment and reactivation have been only partially understood and vary considerably across different cell types, resulting in heterogeneous responses to LRAs (10). Thus, the choice of appropriate *in vitro* models to study the efficacy of selected LRAs in reversing the latency of HIV is critical. Although isolated CD4^+^ T cells provide a model that closely reflects the physiological conditions of HIV infection in humans (56), testing large numbers of compounds directly in primary CD4^+^ T cells could be difficult, especially in the pediatric setting, due to the limited availability of samples from PLWH. In this context, immortalized cell line models, despite not fully recapitulating the immunological and virological complexity observed in PLWH, represent a valuable tool for preliminary screening. Their relatively homogeneous integration pattern and reproducible response enable a direct and quantitative comparison among different compounds, reducing the variability and potential bias associated with the heterogeneous proviral integration landscape characteristic of primary cells. Moreover, immortalized cell lines are easy to maintain over extended periods, allow for the efficient establishment of latent HIV infection, and express high levels of HIV RNA and proteins (10, 57). Despite the use of flow-cytometry, intracellular p24 detection was previously used as an endpoint to quantify HIV reactivation (23–25). The novelty of our two-color flow cytometry protocol is to simultaneously measure p24 and cell viability at single-cell resolution, providing an innovative, standardized, reproducible, easy, low-cost, and time-efficient protocol that is biologically meaningful for the assessment of reactivation while controlling for cytotoxicity. The main objective of this study was to develop a protocol to benchmark different LRAs and ultimately select the most effective compound for HIV reactivation *in vitro* among several selected molecules. By simultaneously assessing viral reactivation through intracellular p24 expression quantification and cell viability using flow cytometry, our approach balances LRA efficacy with manageable toxicity. We initially compared two HIV latency cell lines to identify the most suitable model for evaluating differential levels of HIV latency reactivation. ACH2 cells demonstrated consistently higher levels of both baseline and LRA-induced p24 expression, exhibiting a higher variability, allowing clearer discrimination of compound-specific effects compared to 8E5LAV. Given the primary objective of developing a prescreening protocol to reduce the number of compounds and experimental conditions subsequently tested in patient-derived cells, viability represents a critical parameter. This is especially relevant considering that CD4^+^ T cells are inherently more sensitive to cytotoxic stress, and that selected compounds will ultimately be applied to primary cells destined for downstream sequencing analyses. For this reason, we first excluded the 72-h stimulation time point, as cell viability was below 50% across all conditions. Both 20- and 48-h stimulations resulted in measurable p24 viral reactivation; however, after 48 h, we observed a greater reduction in cell viability. Although it is reported in the literature that some molecules, particularly BET inhibitors, induce the peak of viral reactivation at 48 h (26, 27), confirmed also by our data, the 20-h stimulation still reliably identified the top-performing compounds, preserving a trend comparable to that observed at later time points while providing a substantially improved viability profile. Importantly, this viability trend was consistent across both cell lines and across compounds, indicating that prolonged stimulation itself represents a major source of cytotoxicity. We acknowledge that this represents a compromise between maximal reactivation and cellular health; however, this balance is intentional and central to the proposed pipeline, which is designed to offer a scalable, unbiased prescreening strategy applicable to a large number of LRAs. Importantly, compound-specific optimal conditions can be further explored in subsequent, more targeted studies once promising candidates have been identified. Our protocol allows for the identification of two potent LRAs, PEP005 and CUDC-907, across three independent experimental settings involving different compound and molecule combination strategies. In all instances, these two molecules emerged as the most effective in reactivating latent HIV, suggesting that their mechanism of action may provide a superior reactivation profile compared to other tested agents or combinations. Our analysis of LRA combinations did not reveal consistent improvement in reactivation effects beyond those achieved by the most active single agents. Although selected combinations containing CUDC-907 showed increased p24 levels compared to CUDC-907 alone, these effects were largely driven by the presence of PEP005 and did not exceed the activity observed with PEP005 as a single agent. We subsequently integrated an *in silico* strategy in collaboration with Dompé for the use of the Exscalate platform to further characterize the polypharmacological profiles of PEP005 and CUDC-907 and expand the pool of candidate molecules. This platform overcomes the limitations of the use of virtual screening for binding affinity decoupled from safety profiling and a systemic understanding of the existing platforms by integrating target identification, polypharmacology, and predictive toxicology into a single automated workflow. Through the ProfhEX module, a freely accessible tool, we systematically annotated their predicted interactions across a broad panel of human proteins, identifying additional protein families potentially implicated in latency reversal beyond their primary known targets. We then applied the EXDII module, a proprietary Exscalate tool, for a target expansion approach, retrieving compounds capable of modulating the same protein families. This computational workflow enabled the prioritization of five top-ranking molecules, selected for their predicted activity on the identified targets. The comparison of these new drugs identified Tandutinib as a novel and previously undescribed LRA candidate, positioning it as a promising new compound that warrants further investigation alongside established LRAs. Notably, its prior evaluation in clinical trials supports a favorable safety profile, facilitating its potential repositioning and accelerating its possible clinical translation. In detail, PEP005 acts via the PKC-NF-κB signaling pathway activation, which has been previously shown to efficiently induce HIV transcription in a wide range of latency models (56). Moreover, recent studies demonstrate that LRAs targeting this pathway may be particularly effective at inducing full-length HIV transcription in resting CD4^+^ T cells from individuals on suppressive ART (29, 58). CUDC-907 acts as an inhibitor of both HDAC and phosphatidylinositol-3-kinases. It has been demonstrated that CUDC-907 displayed higher latency-reversal activity than the current most effective HDAC inhibitors tested in anti-HIV-1 trials (romidepsin and panobinostat) without inducing T cell activation or proliferation (56). Tandutinib is a tyrosine kinase inhibitor initially developed for the treatment of hematologic malignancies and has been evaluated in phase I and II clinical trials for acute myeloid leukemia (AML) (59), glioblastoma (60), and metastatic kidney cancer (60), demonstrating modest clinical activity. It selectively targets class III receptor tyrosine kinases, including FLT3, PDGFR, and KIT, which are frequently dysregulated in cancers (59, 61). In this study, we observed that Tandutinib demonstrated an appreciable latency-reversing effect in the ACH2 cell line, despite its known canonical targets not being directly involved in HIV latency reactivation. Moreover, preclinical studies in colon cancer have shown that Tandutinib suppresses phosphorylation of Akt, mTOR, and downstream effectors, such as p70S6K, thereby modulating the PI3K/Akt/mTOR signaling axis (62). This pathway has been implicated in HIV transcriptional regulation and latency maintenance (63); however, inhibition of mTOR signaling has also been reported to impair HIV reactivation in certain experimental systems (63, 64), highlighting the context-dependent nature of its role. Taken together, these observations suggest that the latency-reversing effect observed in our model is unlikely to be explained by a simple linear inhibition of mTOR signaling. Rather, Tandutinib may exert its activity through broader modulation of interconnected host signaling networks or through currently unidentified targets. Further studies in additional latency models and transcriptomics analysis will be required to clarify its mechanism of action and to confirm its potential as a novel LRA. We note that mechanistic investigations on the lead LRAs fall outside the scope of this screening-focused study. Our transcriptomic data on ACH2 cells corroborate the quantification based on p24, showing a marked increase in HIV RNA copies upon stimulation with PEP005 and CUDC-907, supporting the effectiveness of our screening approach in identifying promising latency-reversing compounds. Such in-depth analyses, particularly those involving high-throughput sequencing, are not feasible when testing numerous conditions due to their high costs and complexity. Accordingly, transcriptomic analyses were performed only for PEP005 and CUDC-907, the most potent compounds identified, as a validation of our protocol. This approach will also be applied to Tandutinib in future studies to explore its activity and underlying mechanisms more thoroughly. While we acknowledge that the lack of transcriptomic analyses for all screened compounds represents a limitation of the present study, this choice reflects the sequential design of the proposed workflow. In this context, transcriptomic profiling represents a critical downstream step, enabling not only the confirmation of latency reversal activity but also a comprehensive investigation of mechanisms of action, including known on-target pathways, potential off-target effects, and broader transcriptional changes at the whole-transcriptome level. Accordingly, high-throughput sequencing constitutes the next step of this work and a key future perspective, aimed at strengthening the biological interpretation and translational relevance of the identified compounds. Moreover, the best performing LRAs could also be applied to pediatric samples derived from PHIV individuals, to investigate whether reactivation mechanisms differ in this population and possibly exhibit distinct molecular responses (65). Such information may also provide important knowledge on the reactivation potential of HIV reservoirs, hence informing the eligibility for testing new disease-modifying treatment or analytical treatment interruption studies (66). Nonetheless, it is critical to acknowledge the limitations associated with cell line-based latency models. The reliance on immortalized latency models, in particular the ACH-2 cell line, does not fully capture the complexity of HIV latency *in vivo* or the specific immunological and virological features of HIV infection. Accordingly, the findings reported here should not be interpreted as a direct representation of latency reversal mechanisms in CD4^+^ T cells. Moreover, the ACH2 line, while useful, displays inherent variability in p24 expression and reactivation potential depending on its replication status, which necessitates careful timing and monitoring of experimental conditions. To mitigate these effects and enhance reproducibility, we minimize temporal variability by conducting all experiments within a narrow time frame and by standardizing outcome measures through intra-assay normalization relative to unstimulated controls. These methodological precautions enhanced the robustness and comparability of our data across independent experiments. Moreover, it is increasingly recognized that the molecular mechanisms governing HIV latency and reactivation are highly context-dependent, varying not only between primary and immortalized cells but also among individual latently infected clones (10). This variability contributes to the inconsistent performance of many LRAs across different experimental systems and highlights the necessity of using multiple models to capture the spectrum of latency phenotypes. The poor understanding of the *in vivo* reservoir location and regulatory landscape further complicates the translation of *in vitro* findings into effective clinical interventions. In this context, our study provides a clear and reproducible framework to explore and compare LRA efficacy in a controlled setting, contributing valuable insights into compound prioritization for the following mechanistic characterization in primary cells. Only compounds that demonstrate an optimal balance between latency reactivation and cellular viability in this prescreening phase will be advanced to subsequent validation studies in primary CD4^+^ T cells from PLWH, including pediatric cohorts, where latency reversal will be assessed using transcriptional and sequencing-based readouts. These analyses will follow an established Nanopore sequencing workflow, a technology that is well validated and routinely implemented in our laboratory (49). In conclusion, our study provides a standardized and reproducible framework for the preliminary screening and prioritization of LRAs. By balancing viral reactivation with cell viability and integrating *in silico* profiling, this approach facilitates the identification of promising candidate compounds for downstream mechanistic studies and validation in primary cells. These findings support more efficient and informed strategies for exploring HIV latency reversal.

## Data Availability

All data supporting the findings of this study are included in the article and its supplemental material. Raw sequencing data generated in this study have been deposited in the Gene Expression Omnibus (GEO) database under accession number GSE332561.

## SUPPLEMENTAL MATERIAL

Supplemental figure spectrum.00407-26-s0001.tif Fig. S1

Table S1 spectrum.00407-26-s0002.docx Reagents and consumables.

Table S2 spectrum.00407-26-s0003.xlsx Full list of drugs, including names, chemical structures, and annotations.

ASM does not own the copyrights to Supplemental Material that may be linked to, or accessed through, an article. The authors have granted ASM a non-exclusive, world-wide license to publish the Supplemental Material files. Please contact the corresponding author directly for reuse.

## References

[1] Khoury G, Darcis G, Lee MY, Bouchat S, Van Driessche B, Purcell DFJ, Van Lint C. 2018. The molecular biology of HIV latency. Adv Exp Med Biol 1075:187–212. doi:10.1007/978-981-13-0484-2_8 30030794

[2] Chen J, Zhou T, Zhang Y, Luo S, Chen H, Chen D, Li C, Li W. 2022. The reservoir of latent HIV. Front Cell Infect Microbiol 12:945956. doi:10.3389/fcimb.2022.945956 35967854 PMC9368196

[3] Armani-Tourret M, Bone B, Tan TS, Sun W, Bellefroid M, Struyve T, Louella M, Yu XG, Lichterfeld M. 2024. Immune targeting of HIV-1 reservoir cells: a path to elimination strategies and cure. Nat Rev Microbiol 22:328–344. doi:10.1038/s41579-024-01010-8 38337034 PMC11131351

[4] Angamuthu D, Vivekanandan S, Hanna LE. 2023. Experimental models for HIV latency and molecular tools for reservoir quantification-an update. Clin Microbiol Rev 36:e0001323. doi:10.1128/cmr.00013-23 37966222 PMC10732067

[5] Mbonye U, Karn J. 2024. The cell biology of HIV-1 latency and rebound. Retrovirology (Auckl) 21:6. doi:10.1186/s12977-024-00639-w PMC10996279 38580979

[6] Sengupta S, Siliciano RF. 2018. Targeting the latent reservoir for HIV-1. Immunity 48:872–895. doi:10.1016/j.immuni.2018.04.030 29768175 PMC6196732

[7] Poon B, Grovit-Ferbas K, Stewart SA, Chen IS. 1998. Cell cycle arrest by Vpr in HIV-1 virions and insensitivity to antiretroviral agents. Science 281:266–269. doi:10.1126/science.281.5374.266 9657723

[8] Stewart SA, Poon B, Song JY, Chen IS. 2000. Human immunodeficiency virus type 1 vpr induces apoptosis through caspase activation. J Virol 74:3105–3111. doi:10.1128/jvi.74.7.3105-3111.2000 10708425 PMC111809

[9] Bashiri K, Rezaei N, Nasi M, Cossarizza A. 2018. The role of latency reversal agents in the cure of HIV: A review of current data. Immunol Lett 196:135–139. doi:10.1016/j.imlet.2018.02.004 29427743

[10] Fujinaga K, Cary DC. 2020. Experimental systems for measuring HIV latency and reactivation. Viruses 12:1279. doi:10.3390/v12111279 33182414 PMC7696534

[11] Boehm D, Ott M. 2017. Host methyltransferases and demethylases: potential new epigenetic targets for HIV cure strategies and beyond. AIDS Res Hum Retroviruses 33:S8–S22. doi:10.1089/aid.2017.0180 29140109 PMC5684665

[12] Blazkova J, Trejbalova K, Gondois-Rey F, Halfon P, Philibert P, Guiguen A, Verdin E, Olive D, Van Lint C, Hejnar J, Hirsch I. 2009. CpG methylation controls reactivation of HIV from latency. PLoS Pathog 5:e1000554. doi:10.1371/journal.ppat.1000554 19696893 PMC2722084

[13] Van Lint C, Emiliani S, Ott M, Verdin E. 1996. Transcriptional activation and chromatin remodeling of the HIV-1 promoter in response to histone acetylation. EMBO J 15:1112–1120. 8605881 PMC450009

[14] Ylisastigui L, Archin NM, Lehrman G, Bosch RJ, Margolis DM. 2004. Coaxing HIV-1 from resting CD4 T cells: histone deacetylase inhibition allows latent viral expression. AIDS 18:1101–1108. doi:10.1097/00002030-200405210-00003 15166525

[15] Mbonye U, Karn J. 2011. Control of HIV latency by epigenetic and non-epigenetic mechanisms. Curr HIV Res 9:554–567. doi:10.2174/157016211798998736 22211660 PMC3319922

[16] Bouchat S, Delacourt N, Kula A, Darcis G, Van Driessche B, Corazza F, Gatot J-S, Melard A, Vanhulle C, Kabeya K, Pardons M, Avettand-Fenoel V, Clumeck N, De Wit S, Rohr O, Rouzioux C, Van Lint C. 2016. Sequential treatment with 5-aza-2’-deoxycytidine and deacetylase inhibitors reactivates HIV-1. EMBO Mol Med 8:117–138. doi:10.15252/emmm.201505557 26681773 PMC4734845

[17] Margolis DM. 2011. Histone deacetylase inhibitors and HIV latency. Curr Opin HIV AIDS 6:25–29. doi:10.1097/COH.0b013e328341242d 21242890 PMC3079555

[18] To KKW, Xing E, Larue RC, Li PK. 2023. BET bromodomain inhibitors: novel design strategies and therapeutic applications. Molecules 28:3043. doi:10.3390/molecules28073043 37049806 PMC10096006

[19] Jiang G, Dandekar S. 2015. Targeting NF-κB signaling with protein kinase C agonists as an emerging strategy for combating HIV latency. AIDS Res Hum Retroviruses 31:4–12. doi:10.1089/AID.2014.0199 25287643 PMC4287114

[20] Spivak AM, Planelles V. 2018. Novel latency reversal agents for HIV-1 cure. Annu Rev Med 69:421–436. doi:10.1146/annurev-med-052716-031710 29099677 PMC5892446

[21] Sanz M, Weideman AMK, Ward AR, Clohosey ML, Garcia-Recio S, Selitsky SR, Mann BT, Iannone MA, Whitworth CP, Chitrakar A, Garrido C, Kirchherr J, Coffey AR, Tsai Y-H, Samir S, Xu Y, Copertino D, Bosque A, Jones BR, Parker JS, Hudgens MG, Goonetilleke N, Soriano-Sarabia N. 2023. Aminobisphosphonates reactivate the latent reservoir in people living with HIV-1. Front Immunol 14:1219250. doi:10.3389/fimmu.2023.1219250 37744358 PMC10516574

[22] Exscalate. 2026. AI drug discovery platform. Available from: https://www.exscalate.com/

[23] Cillo AR, Sobolewski MD, Bosch RJ, Fyne E, Piatak M Jr, Coffin JM, Mellors JW. 2014. Quantification of HIV-1 latency reversal in resting CD4+ T cells from patients on suppressive antiretroviral therapy. Proc Natl Acad Sci USA 111:7078–7083. doi:10.1073/pnas.1402873111 24706775 PMC4024870

[24] Passaes CPB, Bruel T, Decalf J, David A, Angin M, Monceaux V, Muller-Trutwin M, Noel N, Bourdic K, Lambotte O, Albert ML, Duffy D, Schwartz O, Sáez-Cirión A. 2017. Ultrasensitive HIV-1 p24 assay detects single infected cells and differences in reservoir induction by latency reversal agents. J Virol 91:e02296-16. doi:10.1128/JVI.02296-16 28077644 PMC5331803

[25] Venkatachari NJ, Zerbato JM, Jain S, Mancini AE, Chattopadhyay A, Sluis-Cremer N, Bar-Joseph Z, Ayyavoo V. 2015. Temporal transcriptional response to latency reversing agents identifies specific factors regulating HIV-1 viral transcriptional switch. Retrovirology (Auckl) 12:85. doi:10.1186/s12977-015-0211-3 PMC4594640 26438393

[26] Zhang X, Lin J, Liang T, Duan H, Tan X, Xi B, Li L, Liu S. 2019. The BET bromodomain inhibitor apabetalone induces apoptosis of latent HIV-1 reservoir cells following viral reactivation. Acta Pharmacol Sin 40:98–110. doi:10.1038/s41401-018-0027-5 29789664 PMC6318340

[27] Liang T, Zhang X, Lai F, Lin J, Zhou C, Xu X, Tan X, Liu S, Li L. 2019. A novel bromodomain inhibitor, CPI-203, serves as an HIV-1 latency-reversing agent by activating positive transcription elongation factor b. Biochem Pharmacol 164:237–251. doi:10.1016/j.bcp.2019.04.005 30991051

[28] Verdikt R, Bendoumou M, Bouchat S, Nestola L, Pasternak AO, Darcis G, Avettand-Fenoel V, Vanhulle C, Aït-Ammar A, Santangelo M, et al.. 2022. Novel role of UHRF1 in the epigenetic repression of the latent HIV-1. EBioMedicine 79:103985. doi:10.1016/j.ebiom.2022.103985 35429693 PMC9038550

[29] Jiang G, Mendes EA, Kaiser P, Wong DP, Tang Y, Cai I, Fenton A, Melcher GP, Hildreth JEK, Thompson GR, Wong JK, Dandekar S. 2015. Synergistic reactivation of latent HIV expression by ingenol-3-angelate, PEP005, targeted NF-kB signaling in combination with JQ1 induced p-TEFb activation. PLoS Pathog 11:e1005066. doi:10.1371/journal.ppat.1005066 26225771 PMC4520526

[30] Gunst JD, Kjær K, Olesen R, Rasmussen TA, Østergaard L, Denton PW, Søgaard OS, Tolstrup M. 2019. Fimepinostat, a novel dual inhibitor of HDAC and PI3K, effectively reverses HIV-1 latency *ex vivo* without T cell activation. J Virus Erad 5:133–137. doi:10.1016/S2055-6640(20)30042-X 31700655 PMC6816120

[31] Lunghini F, Fava A, Pisapia V, Sacco F, Iaconis D, Beccari AR. 2023. ProfhEX: AI-based platform for small molecules liability profiling. J Cheminform 15:60. doi:10.1186/s13321-023-00728-6 37296454 PMC10251600

[32] Life sciences and healthcare. 2026. Clarivate. Available from: https://clarivate.com/life-sciences-healthcare/

[33] Avram S, Wilson TB, Curpan R, Halip L, Borota A, Bora A, Bologa CG, Holmes J, Knockel J, Yang JJ, Oprea TI. 2023. DrugCentral 2023 extends human clinical data and integrates veterinary drugs. Nucleic Acids Res 51:D1276–D1287. doi:10.1093/nar/gkac1085 36484092 PMC9825566

[34] Ursu O, Holmes J, Knockel J, Bologa CG, Yang JJ, Mathias SL, Nelson SJ, Oprea TI. 2017. DrugCentral: online drug compendium. Nucleic Acids Res 45:D932–D939. doi:10.1093/nar/gkw993 27789690 PMC5210665

[35] Gaulton A, Hersey A, Nowotka M, Bento AP, Chambers J, Mendez D, Mutowo P, Atkinson F, Bellis LJ, Cibrián-Uhalte E, Davies M, Dedman N, Karlsson A, Magariños MP, Overington JP, Papadatos G, Smit I, Leach AR. 2017. The ChEMBL database in 2017. Nucleic Acids Res 45:D945–D954. doi:10.1093/nar/gkw1074 27899562 PMC5210557

[36] ChEMBL - ChEMBL. 2026. Available from: https://www.ebi.ac.uk/

[37] Gaunt CM, Rainbow DB, Mackenzie RJ, Jarvis LB, Mousa HS, Cunniffe N, Georgieva Z, Brown JW, Coles AJ, Jones JL. 2021. The MS remyelinating drug bexarotene (an RXR Agonist) promotes induction of human tregs and suppresses Th17 differentiation *in vitro*. Front Immunol 12:712241. doi:10.3389/fimmu.2021.712241 34447379 PMC8382874

[38] Keino H, Watanabe T, Sato Y, Okada AA. 2011. Oral administration of retinoic acid receptor-alpha/beta-specific ligand Am80 suppresses experimental autoimmune uveoretinitis. Invest Ophthalmol Vis Sci 52:1548–1556. doi:10.1167/iovs.10-5963 20861477

[39] Gutierrez L, Jang M, Zhang T, Akhtari M, Alachkar H. 2018. Midostaurin reduces regulatory T cells markers in Acute Myeloid Leukemia. Sci Rep 8:17544. doi:10.1038/s41598-018-35978-0 30510164 PMC6277419

[40] Borges BS, Bueno G de P, Tomiotto-Pellissier F, Figueiredo FB, Soares Medeiros LC. 2022. *In vitro* anti-Leishmania activity of triclabendazole and its synergic effect with amphotericin B. Front Cell Infect Microbiol 12:1044665. doi:10.3389/fcimb.2022.1044665 36699729 PMC9868945

[41] Maxwell JW, Falcinelli SD, Nefedov A, Dorfmeier C, Wu G, Dewey M, Webber AL, Archin NM, Margolis DM, Hazuda DJ, Barnard RJO, Howell BJ. 2020. Cellular gene modulation of HIV-Infected CD4 T cells in response to serial treatment with the histone deacetylase inhibitor vorinostat. J Virol 94:e00351-20. doi:10.1128/JVI.00351-20 32295913 PMC7307144

[42] Telwatte S, Morón-López S, Aran D, Kim P, Hsieh C, Joshi S, Montano M, Greene WC, Butte AJ, Wong JK, Yukl SA. 2019. Heterogeneity in HIV and cellular transcription profiles in cell line models of latent and productive infection: implications for HIV latency. Retrovirology (Auckl) 16:32. doi:10.1186/s12977-019-0494-x PMC6849327 31711503

[43] Emiliani S, Van Lint C, Fischle W, Paras P Jr, Ott M, Brady J, Verdin E. 1996. A point mutation in the HIV-1 Tat responsive element is associated with postintegration latency. Proc Natl Acad Sci USA 93:6377–6381. doi:10.1073/pnas.93.13.6377 8692823 PMC39030

[44] Kim JH, Mosca JD, Vahey MT, McLinden RJ, Burke DS, Redfield RR. 1993. Consequences of human immunodeficiency virus type 1 superinfection of chronically infected cells. AIDS Res Hum Retroviruses 9:875–882. doi:10.1089/aid.1993.9.875 7504936

[45] Bour S, Geleziunas R, Wainberg MA. 1995. The human immunodeficiency virus type 1 (HIV-1) CD4 receptor and its central role in promotion of HIV-1 infection. Microbiol Rev 59:63–93. doi:10.1128/mr.59.1.63-93.1995 7708013 PMC239355

[46] Folks TM, Powell D, Lightfoote M, Koenig S, Fauci AS, Benn S, Rabson A, Daugherty D, Gendelman HE, Hoggan MD. 1986. Biological and biochemical characterization of a cloned Leu-3- cell surviving infection with the acquired immune deficiency syndrome retrovirus. J Exp Med 164:280–290. doi:10.1084/jem.164.1.280 3014036 PMC2188205

[47] Wilburn KM, Mwandumba HC, Jambo KC, Boliar S, Solouki S, Russell DG, Gludish DW. 2016. Heterogeneous loss of HIV transcription and proviral DNA from 8E5/LAV lymphoblastic leukemia cells revealed by RNA FISH:FLOW analyses. Retrovirology (Auckl) 13:55. doi:10.1186/s12977-016-0289-2 PMC4982266 27515378

[48] Rigourd M, Ehresmann C, Parniak MA, Ehresmann B, Marquet R. 2002. Primer unblocking and rescue of DNA synthesis by azidothymidine (AZT)-resistant HIV-1 reverse transcriptase: comparison between initiation and elongation of reverse transcription and between (-) and (+) strand DNA synthesis. J Biol Chem 277:18611–18618. doi:10.1074/jbc.M110836200 11901149

[49] Pascucci GR, Morrocchi E, Pighi C, Rotili A, Neri A, Medri C, Olivieri G, Sanna M, Rasi G, Persaud D, Chahroudi A, Lichterfeld M, Nastouli E, Cancrini C, Amodio D, Rossi P, Cotugno N, Palma P. 2023. How CD4^+^ T cells transcriptional profile is affected by culture conditions: towards the design of optimal *in vitro* HIV reactivation assays. Biomedicines 11:888. doi:10.3390/biomedicines11030888 36979867 PMC10045592

[50] Lehár J, Zimmermann GR, Krueger AS, Molnar RA, Ledell JT, Heilbut AM, Short GF 3rd, Giusti LC, Nolan GP, Magid OA, Lee MS, Borisy AA, Stockwell BR, Keith CT. 2007. Chemical combination effects predict connectivity in biological systems. Mol Syst Biol 3:80. doi:10.1038/msb4100116 17332758 PMC1828746

[51] Geary N. 2013. Understanding synergy. Am J Physiol Endocrinol Metab 304:E237–53. doi:10.1152/ajpendo.00308.2012 23211518

[52] Allegretti M, Cesta MC, Zippoli M, Beccari A, Talarico C, Mantelli F, Bucci EM, Scorzolini L, Nicastri E. 2022. Repurposing the estrogen receptor modulator raloxifene to treat SARS-CoV-2 infection. Cell Death Differ 29:156–166. doi:10.1038/s41418-021-00844-6 34404919 PMC8370058

[53] Lunghini F, Cerchia C, Fava A, Pisapia V, Sacco F, Beccari AR. 2025. ProfhEX: empowering early drug discovery with machine learning-based target profiling and liability prediction. J Chem Inf Model 65:13037–13044. doi:10.1021/acs.jcim.5c02250 41360395 PMC12728921

[54] Archin NM, Bateson R, Tripathy MK, Crooks AM, Yang K-H, Dahl NP, Kearney MF, Anderson EM, Coffin JM, Strain MC, Richman DD, Robertson KR, Kashuba AD, Bosch RJ, Hazuda DJ, Kuruc JD, Eron JJ, Margolis DM. 2014. HIV-1 expression within resting CD4+ T cells after multiple doses of vorinostat. J Infect Dis 210:728–735. doi:10.1093/infdis/jiu155 24620025 PMC4148603

[55] Archin NM, Liberty AL, Kashuba AD, Choudhary SK, Kuruc JD, Crooks AM, Parker DC, Anderson EM, Kearney MF, Strain MC, Richman DD, Hudgens MG, Bosch RJ, Coffin JM, Eron JJ, Hazuda DJ, Margolis DM. 2012. Administration of vorinostat disrupts HIV-1 latency in patients on antiretroviral therapy. Nature 487:482–485. doi:10.1038/nature11286 22837004 PMC3704185

[56] Spina CA, Anderson J, Archin NM, Bosque A, Chan J, Famiglietti M, Greene WC, Kashuba A, Lewin SR, Margolis DM, Mau M, Ruelas D, Saleh S, Shirakawa K, Siliciano RF, Singhania A, Soto PC, Terry VH, Verdin E, Woelk C, Wooden S, Xing S, Planelles V. 2013. An in-depth comparison of latent HIV-1 reactivation in multiple cell model systems and resting CD4+ T cells from aviremic patients. PLoS Pathog 9:e1003834. doi:10.1371/journal.ppat.1003834 24385908 PMC3873446

[57] Rodari A, Poli G, Van Lint C. 2022. Jurkat-derived (J-Lat, J1.1, and Jurkat E4) and CEM-derived T cell lines (8E5 and ACH-2) as models of reversible proviral latency. Methods Mol Biol 2407:3–15. doi:10.1007/978-1-0716-1871-4_1 34985653

[58] Bullen CK, Laird GM, Durand CM, Siliciano JD, Siliciano RF. 2014. New *ex vivo* approaches distinguish effective and ineffective single agents for reversing HIV-1 latency *in vivo*. Nat Med 20:425–429. doi:10.1038/nm.3489 24658076 PMC3981911

[59] DeAngelo DJ, Stone RM, Heaney ML, Nimer SD, Paquette RL, Klisovic RB, Caligiuri MA, Cooper MR, Lecerf J-M, Karol MD, Sheng S, Holford N, Curtin PT, Druker BJ, Heinrich MC. 2006. Phase 1 clinical results with tandutinib (MLN518), a novel FLT3 antagonist, in patients with acute myelogenous leukemia or high-risk myelodysplastic syndrome: safety, pharmacokinetics, and pharmacodynamics. Blood 108:3674–3681. doi:10.1182/blood-2006-02-005702 16902153 PMC1895460

[60] Shepard DR, Cooney MM, Elson P, Bukowski RM, Dreicer R, Rini BI, Garcia JA. 2012. A phase II study of tandutinib (MLN518), a selective inhibitor of type III tyrosine receptor kinases, in patients with metastatic renal cell carcinoma. Invest New Drugs 30:364–367. doi:10.1007/s10637-010-9516-1 20711630

[61] Kottaridis PD, Gale RE, Frew ME, Harrison G, Langabeer SE, Belton AA, Walker H, Wheatley K, Bowen DT, Burnett AK, Goldstone AH, Linch DC. 2001. The presence of a FLT3 internal tandem duplication in patients with acute myeloid leukemia (AML) adds important prognostic information to cytogenetic risk group and response to the first cycle of chemotherapy: analysis of 854 patients from the United Kingdom Medical Research Council AML 10 and 12 trials. Blood 98:1752–1759. doi:10.1182/blood.v98.6.1752 11535508

[62] Ponnurangam S, Standing D, Rangarajan P, Subramaniam D. 2013. Tandutinib inhibits the Akt/mTOR signaling pathway to inhibit colon cancer growth. Mol Cancer Ther 12:598–609. doi:10.1158/1535-7163.MCT-12-0907 23427297 PMC4418457

[63] Crater JM, Nixon DF, Furler O’Brien RL. 2022. HIV-1 replication and latency are balanced by mTOR-driven cell metabolism. Front Cell Infect Microbiol 12:1068436. doi:10.3389/fcimb.2022.1068436 36467738 PMC9712982

[64] Besnard E, Hakre S, Kampmann M, Lim HW, Hosmane NN, Martin A, Bassik MC, Verschueren E, Battivelli E, Chan J, Svensson JP, Gramatica A, Conrad RJ, Ott M, Greene WC, Krogan NJ, Siliciano RF, Weissman JS, Verdin E. 2016. The mTOR complex controls HIV latency. Cell Host Microbe 20:785–797. doi:10.1016/j.chom.2016.11.001 27978436 PMC5354304

[65] Palma P, Foster C, Rojo P, Zangari P, Yates A, Cotugno N, Klein N, Luzuriaga K, Pahwa S, Nastouli E, Gibb DM, Borkowsky W, Bernardi S, Calvez V, Manno E, Mora N, Compagnucci A, Wahren B, Muñoz-Fernández MÁ, De Rossi A, Ananworanich J, Pillay D, Giaquinto C, Rossi P. 2015. The EPIICAL project: an emerging global collaboration to investigate immunotherapeutic strategies in HIV-infected children. J Virus Erad 1:134–139. doi:10.1016/S2055-6640(20)30510-0 26893908 PMC4755515

[66] Kuhn L, Barnabas S, Cotugno N, Peay H, Goulder P, Cotton M, Violari A, Pahwa S, Reddy K, Tagarro A, et al.. 2024. Analytical treatment interruption in children living with HIV: position statement from the EPIICAL consortium. Lancet HIV 11:e700–e710. doi:10.1016/S2352-3018(24)00157-7 39059402 PMC12971027

