# Supplementary material for: Selection of effective LRAs using a newly designed in vitro HIV latency reactivation protocol: toward future application in HIV samples
Source: Microbiol Spectr. 2026 Aug 4;14(9):e00407-26. doi: 10.1128/spectrum.00407-26 (PMC13532288; doi:10.1128/spectrum.00407-26)
Supplement: Supplementary file 1 [file spectrum.00407-26-s0002.docx]

**Supplementary Table 1**

**REAGENTS AND CONSUMABLES**

| **Reagent name and Consumables** | **Catalog and brand** |
| --- | --- |
| ACH2 Cell Line | Repository reference: ARP138, code 0138 |
| 8E5/LAV Cell Line | Repository reference: ARP110, code 0110 |
| Dulbecco's Phosphate Buffer Saline (PBS) | Cat #ECB4004L, Euroclone |
| RPMI | Cat. #ECM9106L, EuroClone |
| EDTA | Cat #324506-100, Merck |
| Lookout® One-Step Mycoplasma PCR Detection Kit | Cat #MP0050-100TST, Sigma-Aldrich |
| Fetal Bovine serum (FBS) | Ref. #10270-106, Gibco, ThermoFisher Scientific |
| Dimethyl Sulfoxide (DMSO) | Cat #D8418-250, Sigma Aldrich |
| Paraformaldehyde (PFA) | Cat #158127-500G, Sigma Aldrich |
| L-Glutamine | Cat. #ECB3000D, EuroClone |
| Penicillin/Streptomycin solution (Pen/Strep) | Cat. #ECB3001D, EuroClone |
| Ficoll-Paque PLUS | Cat. #17144003, Cytiva Sweden AB, SE-751 84 |
| Trypan Blue Solution | Cat. #T8154 Sigma-Aldrich, Merck KGaA |
| p24 Antigen Stain | Cat. #6604667, Beckman Coulter |
| PMA | Cat. #P1585, Sigma-Aldrich, Merck KGaA |
| Ionomycin | Cat. #I0634, Sigma-Aldrich, Merck KGaA |
| IL-2 (Interleukin-2) | Cat. #200-02, Peprotech |
| AZT (Azidothymidine) | Cat. #3485, National Institutes of Health, NIH, HIV Research Program, HRP, Bethesda, MD, USA |
| Beta-mercaptoethanol (BME) | Ref #21985-023, Gibco, ThermoFisher Scientific |
| BD Perm solution II | Cat. #347692, Dickinson and Company BD Biosciences |
| BD Horizon™ Fixable Viability Stain 510 | Cat. #564406, Dickinson and Company BD Biosciences |
| EasySep Human CD4⁺ T Cell Enrichment Kit | Cat. #19052, Stemcell Technologies |
| EasySep™ D Magnetic Particles | Cat. #17952, Stemcell Technologies |
| EasySep™ Human CD4⁺ T Cell Isolation Cocktail | Cat. #17952C, Stemcell Technologies |
| Nuclease-free water | Cat. #AM9937, ThermoFisher |
| UltraPure™ DNase/RNase-Free Distilled Water | Cat. #10977035, ThermoFisher |
| Falcon Tubes 5 mL Polystyrene round-bottom tube | Cat. #352054, Corning |
| 5 ml Eppendorf | Cat. #022510509, Eppendorf |
| 5 mL Polystyrene round-bottom tube | Cat. #352054, Falcon/SIAL |
| 200 µl Ultra Point graduated tips refill | Ref. #S1113-1700, Tipone |
| Serological Tips 5 ml, 10 ml | Ref. #4488; 4487, Costar |
| Countess II | ThermoFisher Scientific |
| RNeasy Lysis Buffer (RLT Buffer) | Cat #1053393 QIAGEN |
| Beta-mercaptoethanol (BME) | Ref. #21985-023, Gibco, ThermoFisher Scientific |
| AllPrep DNA/RNA/Protein Mini Kit™ and AllPrep DNA/RNA/Protein Micro Kit™ | Cat. #80204 and #:80284 respectively, QIAGEN |
| RNase-free water | Mat. #1039480, QIAGEN |
| SUPERase·In™ RNase Inhibitor | Cat. #AM2694, Invitrogen, ThermoFisher Scientific, |
| PCR-cDNA Barcoding kit SQK-PCB109 | Oxford Nanopore Technologies |
| Qubit™ RNA HS Assay Kit | Cat. #Q32852, Invitrogen |
| Qubit™ 4 Fluorometer | Cat. #Q33238, Invitrogen |
| Apabetalone | Cat. #CS-3239, ChemScene |
| Ingenol-3-angelate | Cat. #SML1318-1MG, Sigma Aldrich |
| (−)-Epigallocatechin gallate | Cat. #E4268-100MG, Sigma Aldrich |
| CPI203 | Cat. #SML1212-5MG, Sigma Aldrich |
| 5-Aza-2′-deoxycytidine | Cat. #A3656-5MG, Sigma Aldrich |
| Alendronate | Cat. #126855-100MG, Sigma Aldrich |
| CUDC-907 | Adooq Bioscience, #A11153 |
